# Starting Digital Treatment for Neurodevelopmental Disorders by Experience Experts: On the Waiting List for Diagnostic Assessment via the Super Brains App

**DOI:** 10.1192/j.eurpsy.2022.148

**Published:** 2022-09-01

**Authors:** J.J.S. Kooij, R. Den Hollander

**Affiliations:** Super Brains BV, Oud Beijerland, Netherlands

**Keywords:** adhd, experience experts, digital treatment, waitinglist

## Abstract

Digital treatment for neurodevelopmental disorders is being developed in order to treat patients online when possible, to reduce waitinglists, and to improve efficiency of treatment. In this workshop, first experiences with the so called Start Programme of the Super Brains app for ADHD in adults are presented. The Super Brains app has been developed by Rutger den Hollander, who himself has ADHD and owns an ICT company in the Netherlands, in cooperation with the speaker of this workshop and Parnassiagroep. The Start Programme is a new part of the Super Brains app, meant for patients referred for treatment that have to wait on often long waitinglists. Now they have no longer to wait, but can start immediately preparing for assessment by filling in questionnaires, and with psycho-education, lifestyle tips and support by experience experts that welcome them in the app and show them around. First data on the use of different parts of the Start Programme, the activity of patients in the app, and the satisfaction of patients will be presented. We also aim to study whether the Start Progamme is effective in reducing severity of ADHD symptoms. Super Brains can be adjusted for use in patients with other (neurodevelopmental) disorders easily.

**Disclosure:**

No significant relationships.

